# Correlates of dual/poly tobacco use among school-going adolescents in Malaysia: Findings from a nationwide school-based study

**DOI:** 10.18332/tid/148247

**Published:** 2022-06-02

**Authors:** Kuang Hock Lim, Yoon Ling Cheong, Hui Li Lim, Sumarni Mohd Ghazali, Chee Cheong Kee, Yong Kang Cheah, Pei Pei Heng, Mohd Hazilas Mat Hashim, Cia Vei Tan, Jia Hui Lim

**Affiliations:** 1 Institute for Medical Research, National Institutes of Health, Kuala Lumpur, Malaysia; 2 Clinical Research Centre, Hospital Sultan Ismail, Johor Bahru, Malaysia; 3 Department of Biostatistics and Data Repository, National Institutes of Health, Shah Alam, Malaysia; 4 School of Economics, Finance and Banking, Universiti Utara Malaysia, Sintok, Malaysia; 5 Pharmacy Department, Hospital Putrajaya, Putrajaya, Malaysia

**Keywords:** cigarette, e-cigarette, shisha, school-going adolescents, dual/poly user

## Abstract

**INTRODUCTION:**

Dual/poly tobacco use is common among youths globally. However, in Malaysia information on dual/poly tobacco use is scarce, thus the present study examines the prevalence and factors associated with dual/poly tobacco users among school-going adolescents in Malaysia.

**METHODS:**

We derived data on tobacco and e-cigarette use among Malaysian adolescents from a nationwide school-based study conducted in 2016. A total of 13135 adolescents responded in the cross-sectional survey which used multi-stage sampling to select a representative sample of school-going adolescents aged 11–19 years. A standard validated questionnaire was used to obtain the data and multiple logistic regression was conducted to assess factors associated with dual/ poly tobacco use.

**RESULTS:**

The prevalence of dual/poly tobacco use was 6.5%, more than half of which were both conventional and e-cigarette users. Multivariable logistic regression revealed that the likelihood of dual tobacco use was significantly higher among males (AOR=14.73; 95% CI: 9.11–23.81), secondary school students, those aged 16–19 years (AOR=5.99; 95% CI: 4.04–8.87), natives of Sabah (AOR=7.41; 95% CI: 3.48–15.79), and those never been taught on the health hazards of tobacco at school, exposed to secondhand smoke (SHS) at home, school or other public areas, and had a positive perception of e-cigarettes and lower perception of the harms of tobacco smoking.

**CONCLUSIONS:**

Although the prevalence of dual/poly users was still low among Malaysian school-going adolescents, proactive measures should be taken to reduce dual tobacco use among youth in Malaysia with focus on the factors identified in this study.

## INTRODUCTION

Smoking-related illnesses have been a significant contributor to preventable diseases and deaths in Malaysia over the past few decades^1^. This is expected to continue given the current trend, i.e. plateau in adult smoking prevalence, with small reduction in teen smoking prevalence over the past decade^2^. Studies have shown that smoking is a behavior initiated during adolescence^3^. Adolescents display evidence of addiction at much lower levels of tobacco consumption compared to adults, and smoking initiated in adolescence results in high level of nicotine addiction and lower quit success rates^4,5^. Those who do not smoke as adolescents generally do not start smoking as adults^5^. Therefore, reducing the incidence of smoking among adolescents is one of the long-term measures to reduce the prevalence of smoking in the population^6^.

Following the introduction of some tobacco products such as shisha (waterpipe or hookah) and e-cigarettes in the market, efforts to reduce smoking among the population in the country have become more difficult. Available in an array of flavors, colors and packaging, they have become a big attraction among youths^7,8^. As a result, there has been promotion of tobacco products among smokers, non-smoking adolescents’ trial and initiation of smoking, and a potential increase in the number of dual (smokers using of two types of tobacco product) or poly tobacco users (using three or more types of tobacco products). Globally, it is estimated that more than 20% of smokers aged ≥15 years use cigarettes and at least one other tobacco product concurrently^9^. In the United States, it was estimated that 4.6 million middle and high school students were current users of tobacco products (i.e. have used a tobacco product in the past month) in 2014, 2.2 million of whom used at least two types of products^10^. Studies showed poly users were more likely to be male^11^, Caucasian^11^, had more exposure to and were more receptive to tobacco advertising^12^, more exposed to secondhand smoke and have lower perception of harm of smoking and SHS^13^. However, with respect to urban and rural areas there was no significant association between locality with dual/poly tobacco product use^14^.

Studies have found that smokers who use two or more tobacco products are more vulnerable to nicotine addiction^15^. Users of two or more types of tobacco products were also found to have a higher risk of relapse 30-days post-cessation compared to single users^15^, especially among adolescents^16^, as well as higher risk of using other substances such as alcohol, cannabis, marijuana or other illicit drugs^17^. Dual/poly tobacco product use is linked to increased exposure to toxicants and carcinogens^18^. As a result, dual/poly tobacco use may subsequently result in an increased risk of tobacco-related morbidity and mortality.

Although studies on cigarette and other alternative products in the country are abundant, data on dual/ poly tobacco use among adolescents are lacking as previous studies reported the prevalence and factors related to cigarette smoking^3^ and e-cigarettes^19^ use individually. This information is crucial to support policy efforts and interventions to curb the use of tobacco products among adolescents in the country. The aim of this study is to determine the prevalence and factor(s) associated with dual/poly tobacco use among school going adolescent in Malaysia.

## METHODS

### Sample and data

We analyzed data from the Tobacco and E-Cigarette survey among Malaysian Adolescents (TECMA) conducted in 2016, which was a large nationally representative survey that collected data on tobacco smoking, e-cigarette use, and shisha use, among public primary and secondary school-going adolescents aged 11–19 years. Multistage cluster sampling was used to select a sample of students aged 11–19 years. Fifteen states in Malaysia were the first strata, while urban and rural areas within each state in Malaysia were the second strata. Schools were the primary sampling unit selected, which were based on the proportion to the school’s enrollment size for the survey year and the latest sampling frame provided by the Ministry of Education Malaysia. The sample selection was followed by the selection of classes in selected schools using the simple random sampling method. Finally, all the school-going adolescents in the selected class were invited to participate in the study. The sample size was 13980 based on the estimated prevalence of 3% of e-cigarette users, type one error (alpha of 5%), design effect of 3 to cater for clustering effect, a tolerable error of 1.5% and expected non-response rate of 20%.

### Data collection

Only students with written consent from parents were included as participants in the study. Data collection was carried out in a designated area identified by school administrators during the school day. No school teachers or staff was present during the data collection to avoid the Hawthorne effect, i.e. the tendency of research subjects to act atypically as a result of their awareness of being studied. To ensure response validity, the questionnaires were self-administered and assurances of respondent anonymity and data confidentiality were given. Trained research team members briefed the respondents in detail about the items in the questionnaire, and team members were at hand to provide clarification on any of the items when needed. Respondents were also told that their participation was voluntary and could skip any of the items in the survey. In addition to obtaining guardian/ parental consent, selected respondents signed an assent form before participating in the study.

### Questionnaire

The questionnaire used in the TECMA study was adopted from the Global Youth Tobacco Survey (GYTS) 2003, GYTS 2009 and Global School Health Survey (GSHS) 2012 in Malaysia, and had been validated. To establish the face-validity of the questionnaire, the questionnaire was pretested on thirty students from primary and secondary schools in Kuala Lumpur to ensure that the items suited the local sociocultural context. Minor modifications were made to the questionnaire based on the feedback from the pre-test.

### Dependent variables

*Non-use, mono, and dual/poly use of tobacco products*

Classification of non-use, mono use and poly tobacco use was based on the frequency of using three types of tobacco products (cigarettes, shisha and e-cigarettes) in the past one month. Those who used any of the products for at least one day in the past month were categorized as current users of that product. Respondents who answered ‘yes’ to items using tobacco products were assigned a value of 1, while those who answered ‘no’ were assigned a value of 0. We totaled the number of tobacco products they were currently using (range: 0–3). Students were classified as: 1) non-users if they did not use any tobacco products (score=0); 2) mono users if they only used one type of tobacco product (score=1); and 3) dual/poly users if they used two or more types of tobacco products (score=2–3).

### Independent variables

The independent variables in the study were sociodemographic variables, namely gender, age (i.e. aged ≤12, 13–15, and 16–19 years), ethnicity (i.e. Malay, Chinese, Indian, Bumiputra Sabah, Bumiputra Sarawak, and other), locality of schooling (i.e. urban or rural), ever been taught at school about the dangers of tobacco product, exposure to secondhand smoke (SHS) at school/home/public areas - other than home, perceived the hazardous health effects of tobacco, and ever exposed to tobacco promotion at point of sale.

### Statistical analysis

*Data management and analysis*

Data were keyed in twice by two data entry teams (double-entry) as a quality control measure. Discrepancies between the datasets were resolved by referring to the original questionnaire. The data were cleaned, and design weights based on the study design and sampling method and post-stratification weights to adjust for non-response were calculated for each case in the dataset and applied during analysis. Descriptive statistics were used to describe the demographic characteristics of the respondents. The chi-squared test was used to determine the association between mono use, and dual/poly use, with each categorical independent variable. Multivariable multinomial regression (MMR) was conducted to determine factors associated with mono use and dual/poly use. The data were presented as non-user versus mono user, and non-user versus dual/poly user. Independent variables with p=0.25 or less that were in univariate analysis were included in the model to determine the effect of each on the dependent variable (i.e. poly user of the tobacco product) after adjusting for the influence of other independent variables. Subsequently, all two-way interactions between independent variables in the final models were also examined, p>0.05 indicated that there were no significant two-way interactions. The overall fit of the MMR model was satisfactory, with no outliers or multicollinearity. All statistical analyses were performed at the 95% confidence level using SPSS software version 24.

### Definition of tobacco users

Tobacco users were defined as those who had used any tobacco product (cigarettes, e-cigarettes, or shisha) at least once in the previous 30 days.

## RESULTS

### Sample characteristics

The response rate was 88.7% (n=13162/14832). Participants consisted of school-going adolescents aged 11–19 years. The distribution of respondents by gender was almost equal, i.e. male 51.1% and female 48.9%. Approximately two-thirds of respondents were Malay, the remaining consisting of Chinese (13%), and Sarawak, Sabah Native and Indian ethnicity (5.4– 5.7%). More than one-third of respondents were aged ≤15 years, and more than half (54.5%) were in rural areas (Table 1).

**Table 1:** Characteristics of the respondents who participated in the TECMA Study 2016 among school-going adolescents in Malaysia (N=13136)

*Variables*	*Estimated population*	*n*	*%*
**Gender**			
Male	1881131	6582	51.1
Female	1803629	6554	48.9
**Age** (years)			
≤12	1369393	4138	37.2
13–15	1434842	5278	38.9
16–19	880523	3720	23.9
**Ethnicity**			
Malay	2433432	9243	66.1
Chinese	477966	1764	13.0
Indian	213674	748	5.8
Sabah native	211781	545	5.7
Sarawak native	199558	447	5.4
Other	147095	385	4.0
**Locality**			
Urban	1677958	7685	45.5
Rural	2006801	5448	54.5

Among the students, 14.5 % were tobacco users, while 8.0 % and 6.5 % were mono and dual/poly users, respectively. A significantly higher proportion of mono and dual/poly tobacco product users was observed among males, older age, Malay, Sabah and Sarawak natives, those who perceived less harm in tobacco product usage, not taught in school and exposed to SHS at home, outside the home and at school. In addition, the proportion of ever exposure to smoking promotion at the point of sale was also higher among mono and dual/poly tobacco users (Table 2).

**Table 2:** Prevalence of non-tobacco product users, mono tobacco product users and dual/poly users among school-going adolescents in Malaysia who participated in the TECMA study (2016) (N=13056)

*Variables*	*Non-tobacco product user*			*Mono tobacco product user*			*Dual/poly tobacco product user*			*p*
	*Estimated population*	*n*	*% (95% CI)*	*Estimated population*	*n*	*% (95% CI)*	*Estimated population*	*n*	*% (95% CI)*	
**Gender**										
Male	1390475	4877	73.9 (72.3–75.4)	296706	1013	15.8 (14.5–17.1)	193948	612	10.3 (9.3–11.4)	<0.001
Female	1704942	6290	95.6 (94.8–96.4)	53111	191	2.9 (2.4–3.6)	25575	73	1.4 (1.0–2.1)	
**Age** (years)										
≤12	1244606	3745	90.9 (89.7–91.9)	90319	281	6.6 (5.7–7.6)	34467	112	2.5 (2.0–3.2)	<0.001
13–15	1162810	4414	81.0 (79.2–82.8)	160169	530	11.2 (9.8–12.7)	111863	334	7.8 (6.6–9.2)	
16–19	708001	3008	80.4 (78.5–82.2)	99329	393	11.6 (9.8–12.9)	73193	319	8.3 (7.2–9.6)	
**Ethnicity**										
Malay	2003150	7675	82.3 (81.0–83.5)	258373	943	10.6 (9.7–11.7)	171909	625	7.1 (6.3–8.0)	<0.001
Chinese	452681	1678	94.7 (92.9–96.1)	18369	55	3.8 (2.6–5.5)	6915	31	1.4 (0.9–2.2)	
Indian	195041	679	91.3 (88.6–93.4)	12052	48	5.6 (4.0–7.9)	8580	21	3.1 (1.9–5.1)	
Sabah native	172748	438	81.6 (77.4–85.1)	22011	56	10.4 (7.9–13.5)	17021	41	8.0 (5.5–11.7)	
Sarawak native	163530	367	81.9 (77.6–85.6)	24371	55	12.2 (9.2–16.0)	11656	25	5.8 (3.6–8.9)	
Other	127015	326	86.3 (81.7–89.9)	14640	37	10.0 (6.8–14.4)	5439	62	3.7 (2.3–6.0)	
**Locality**										
Urban	1473469	8656	87.8 (86.9–88.9)	125790	610	7.5 (6.7–8.3)	78698	422	4.7 (4.1–5.3)	<0.001
Rural	1641948	4511	81.8 (80.3–83.3)	204027	594	11.2 (10.0–12.4)	140825	343	7.0 (6.1–8.0)	
**School-based education of tobacco harms**										
Yes	2177172	8052	87.5 (86.5–88.5)	200781	711	8.1 (7.3–9.0)	108981	386	4.4 (3.8–5.1)	
No	182178	812	82.4 (78.3–85.8)	27088	83	12.3 (9.3–15.9)	11816	41	5.3 (3.5–8.0)	<0.001
**SHS exposure at home**										
Yes	1047380	3513	75.2 (73.3–77.0)	197458	852	14.2 (12.8–15.7)	148169	479	10.6 (9.3–12.1)	<0.001
No	2067315	7652	90.2 (89.2–91.2)	152358	552	6.7 (5.9–7.5)	71354	286	3.1 (2.6–3.7)	
**SHS exposure at public areas**										
Yes	1449229	5159	769 (75.3–78.4)	258452	885	13.7 (12.5–15.0)	178250	635	9.4 (8.4–10.6)	<0.001
No	1864349	6004	92.6 (91.6–93.7)	91365	318	5.1 (4.4–5.9)	41273	130	2.3 (1.8–2.9)	
**SHS exposure at school**										
Yes	742137	2767	74.0 (71.9–74.0)	143030	505	14.3 (12.7–18.0)	117460	442	11.7 (10.3–13.3)	<0.001
No	2369786	8390	88.5 (87.5–89.5)	205838	694	7.7 (6.9–9.6)	101025	321	3.8 (3.2–4.5)	
**Saw tobacco advertisement at point of sale**										
Yes	1134356	4038	82.3 (80.7–83.9)	140537	510	10.2 (9.0–11.5)	102846	366	7.5 (6.5–8.6)	<0.001
No	1981061	7139	85.9 (84.7–87.0)	209280	694	9.1 (8.2–10.1)	116679	399	5.1 (4.3–5.9)	

Approximately 3 in 5 dual/poly tobacco users used e-cigarettes first before using other tobacco products, 26.5% used cigarettes, and 11.1% used shisha first before moving to other tobacco products. Table 3 shows that more than half of dual/poly users used e-cigarettes and cigarettes (53.4%), and more than 1 in 5 respondents used all tobacco products (i.e. cigarettes, e-cigarettes and shisha), while more than two-fifths of dual/poly users used e-cigarettes and shisha, or shisha and cigarettes.

**Table 3:** Type of tobacco products first used and type of tobacco product used by dual/poly tobacco users in the TECMA Study (2016) in Malaysia (N=750)

*Variables*	*Estimated population*	*Sample*		*95% CI*	
		*n*	*%*	*Lower*	*Upper*
**Type of tobacco product first used**					
E-cigarette	139409	491	62.5	56.8	67.8
Cigarette	59047	193	26.5	21.5	32.0
Shisha	24755	66	11.1	8.1	15.0
**Type of tobacco product used**					
Cigarette and e-cigarette	117941	403	53.7	48.3	59.1
Cigarette and shisha	25836	99	11.8	8.7	15.8
E-cigarette and shisha	23682	89	10.8	8.0	14.4
Cigarette, e-cigarette and shisha	52063	174	23.7	19.4	28.7

Multivariable logistic regression revealed that the odds of dual/poly use were significantly higher among males (AOR=14.73; 95 % CI: 9.11–23.81), Sabah and Sarawak natives (AOR=7.41; 95% CI: 3.48–15.79; AOR=3.27, 95% CI: 1.43–7.44, respectively), aged 13–15 years (AOR=3.50; 95% CI: 2.45–5.29) and aged 16–19 years (AOR=5.99; 95% CI: 4.04–8.87), perceived less health harmful effect of smoking (AOR=2.98; 95% CI: 2.15–3.85) and never been taught the health hazard of tobacco products in school (AOR=2.24; 95% CI: 1.45–3.46). However, a comparison between mono and dual/poly users revealed that only older age groups were significantly associated with higher odds of becoming dual/poly users (Table 4).

**Table 4:** Multinomial logistic regression and the associated factor(s) of becoming dual/poly user among school-going adolescents aged 11–19 years who participated in the TECMA study 2016 in Malaysia

*Variables*	*Mono user vs non-user*	*Dual user vs non-user*
	*AOR (95% CI)*	*AOR (95% CI)*
**Gender**		
Male	9.30 (6.04–10.42)	14.73 (9.11–23.81)
Female (Ref.)	1	1
**Age** (years)		
≤12 (Ref.)	1	1
13–15	1.99 (1.52–2.60)	3.50 (2.45–5.29)
16–19	1.98 (1.50–2.67)	5.99 (4.04–8.87)
**Ethnicity**		
Malay	3.75 (2.28–6.14)	5.93 (3.31–10.61)
Chinese (Ref.)	1	1
Indian	1.41 (0.72–2.71)	2.22 (0.96–5.16)
Sabah native	3.73 (2.01–6.91)	7.41 (3.48–15.79)
Sarawak native	3.71 (1.98–6.94)	3.27 (1.43–7.44)
Other	3.82 (1.84–7.91)	4.21 (1.80–49.88)
**Locality**		
Urban (Ref.)	1	1
Rural	1.33 (1.07–1.66)	1.06 (0.82–1.31)
**Smoking harmful**		
Yes (Ref.)	1	1
No	1.76 (1.39–2.23)	2.98 (2.15–3.85)
**School-based education of tobacco harms**		
Yes (Ref.)	1	1
No	1.83 (1.34–2.49)	2.24 (1.45–3.46)
**SHS exposure at school**		
Yes	1.79 (1.41–2.27)	2.59 (1.95–3.43)
No (Ref.)	1	1
**SHS exposure at public areas**		
Yes	2.03 (1.54–2.68)	2.49 (1.71–3.60)
No (Ref.)	1	1
**SHS exposure at home**		
Yes	1.90 (1.48–2.45)	2.45 (1.43–3.53)
No (Ref.)	1	1
**Expose to tobacco promotion at the point of sale**		
Yes	0.97 (0.77–1.23)	1.03 (0.78–1.36)
No (Ref.)	1	1

## DISCUSSION

This is the first study that investigated the prevalence and factors associated with dual/poly tobacco use among school-going adolescents in Malaysia. The study revealed that 6.5% of school-going adolescents used dual/poly tobacco products. The prevalence is significantly lower than the 19.1% prevalence of poly use reported among high school students in the USA^20^, and 20% globally^9^. However, it is almost similar to the 5.4% reported by Aleyan et al.^21^ and 7.0% Cooper et al.^22^ among youths in Canada and Texas, USA, respectively.

Conventional cigarettes and e-cigarettes were the most common products used by dual/poly users in this study. This finding is consistent with another study^23^. This finding might be due to the easy accessibility to these two products^24^. Cigarettes are widely available, widely used and have been in the market for a long time. For instance, cigarettes are sold in sundry shops, service stations and other premises in the country. At the same time, e-cigarettes are also available in premises selling vape/e-cigarette or online, compared to shisha, which are limited and are only available in certain types of premises. Therefore, putting restriction on adolescents to prevent them from patronizing these premises may reduce their usage of shisha among adolescents.

Most dual/poly users of tobacco products used e-cigarettes first and then started using conventional cigarettes or shisha. This is consistent with findings of a longitudinal study that reported that e-cigarette users were 6.17 and 4.98 times more likely to begin using cigarettes and combustible tobacco products (including hookah, cigars, or pipes), respectively^25^.

Many studies among adolescents, mainly in the US, have shown a higher prevalence of dual/poly tobacco use among males^22^. Our study had the same finding. Among the plausible reasons is that smoking is the norm among males in Malaysia. Many studies have shown that the smoking prevalence is significantly higher among adult and youth males in Malaysia. Lim et al.^26^ reported that the smoking rate among adult males was significantly higher compared to females [48.2% in males (95% CI: 47.1–49.4) vs 3.4% (95% CI: 3.1–3.8) in females, in 1996, 46.4% (95% CI: 45.5–47.4) vs 1.60% (95% CI: 1.5–1.8) in 2006, 43.9% (95% CI: 41.4–44.6) vs 1.00 (95% CI: 0.7–1.6) in 2011, and 43.0% (95% CI: 41.4–44.6) vs 1.40 (95% CI: 1.10–1.18) in 2015]. In addition, comparison of male and female youth (aged 13–15 years) status of current smoking from three nationwide cross sectional studies in 2003, 2009 and 2016 revealed a similar gender disparity among Malaysian youth, i.e. significantly higher among males, i.e. 26.1% (95% CI: 19.9–33.4) vs 2.4% in females (95% CI: 1.4–4.1) in 2016, 30.9% (95% CI: 29.6–33.3) vs 6.1% (95% CI: 4.6–7.4) in 2009, and 35.5% (95% CI: 33.2–38.0) vs 4.3% (3.4–5.4) in 2003. Therefore, the use of any tobacco product or other form of tobacco product by this group is the norm. Moreover, Malaysian society still holds on to traditions wherein the social norms governing female behavior may attribute to the reduced involvement of female respondents in smoking cigarettes and other tobacco products^2^.

The prevalence and odds of being dual/poly users of tobacco products were higher among respondents from older age groups (i.e. aged ≥16 years). The finding is consistent with various studies. For example, Willis et al.^27^ reported prevalence of dual/poly users of tobacco products were almost two times higher among adolescents in 10th grade compared to those in 9th grade. In addition, Osman et al.^28^ also reported a positive linear correlation between age and prevalence of usage, whereby 11% among adolescents aged 14 years were tobacco product dual/poly users, but the prevalence increased to 22% among adolescents aged 15 and 16 years, and further increased to 45% among those aged 17 years. This finding might be due to the majority of mono smokers (cigarette smokers) being among the older aged youths. Kowitt et al.^20^ found that susceptibility and tendency to use other tobacco products are among mono users of cigarettes. In addition, parents/guardians in Asian countries, including Malaysia, might give more protection to younger adolescents than to those from older age groups. The freedom enjoyed by older adolescents may increase the likelihood of involvement in risky health behaviors such as smoking. However, this needs to be investigated further in future studies.

We also found differences in the prevalence and odds of being mono or dual/poly users between ethnic groups and schooling locality. For instance, high prevalence and odds of mono use of tobacco products were reported among Malay, Sabah and Sarawak native adolescents, as well as in the rural areas. The odds of being dual/poly tobacco users were significantly higher among native Sabahan youths. This finding is interesting, as it could be because e-cigarettes and shisha are new products compared to cigarettes, therefore, they are less widely used. This is further strengthened by the low prevalence of their use among all youths from different ethnic groups and schooling areas. This suggests there is an opportunity to formulate and implement appropriate policies or measures to stem the use of these products, which in turn reduces the incidence and prevalence of dual/ poly tobacco use among youths in Malaysia.

Exposure to SHS at home, school and outside the house increased the odds of being a dual/poly user of tobacco products. The finding is congruent with the study of Mamudu et al.^29^ among youth in Tennessee. Exposure to tobacco use at home, school and outside the house increases tobacco use among youth because it normalizes tobacco use and makes the behavior socially acceptable. These might be among the plausible reasons for the findings obtained in this study. Thus, smoke-free home and/or vehicle policies would be required to reduce youth exposure and the negative implications of social acceptance of tobacco use. This affirms the US Surgeon General’s suggestion that the coverage of smoke-free policies should be extended to include e-cigarettes^30^.

The prevalence and the odds of being a dual/ poly user were significantly lower among those who perceived tobacco use to have adverse health effects. The finding was in-line with Osman et al.^28^ who reported 1.79 times higher odds of being a dual/poly user of tobacco among respondents who perceived tobacco use to be less harmful. In addition, the finding is also consistent with findings by Cooper et al.^22^ among youth in the US. This finding may be explained by the Health Belief Model^31^ that states that an individual who perceives the severity of behavior will stay away from the behavior. It is also in line with the Cognitive Dissonance Theory, in that people who want to persist with a negative behavior will tend to downplay the adverse effects to rationalize the behaviour^32^.

Exposure to tobacco product advertisements at point of sale was found to be insignificantly associated with mono and dual/poly use. This observation may be because these advertisements affect those who have yet to smoke more, making them susceptible to initiate smoking, as reported by an international study^33^. However, future studies need to examine whether the findings in this study are consistent for adolescents in this country and understand the cognitive mechanism behind it among dual/poly tobacco users.

In this study, we found that mono and poly users shared similar characteristics. This result has also been reported by a study in the US^34^. Other studies have also shown that mono users have a higher likelihood of becoming poly users^22^. Thus, it is imperative to identify budding smokers and initiate preventive measures to reduce smoking among youths.

### Limitations

The study has several limitations. Firstly, as the data on tobacco use were self-reported, this may cause recall or response bias. Secondly, several variables such as drinking habit^35^ and peer smoking^35^, which had shown significant association with dual/poly users, was not investigated in this study. Thirdly, the cross-sectional study design does not allow the causal relationship to be established between independent and dependent variables. Fourth, only three types of tobacco products were investigated in the current study; this might underestimate the actual dual/poly users of tobacco products among school-going adolescents in Malaysia. Lastly, there is no biochemical validation to verify smoking status in this study. Therefore, there may have been under-reporting of tobacco use. Fifth, since the data were collected in 2016, they might not reflect the current situation of dual/poly use of tobacco among youth in Malaysia. However, a previous study in Malaysia showed the consistency between self-reported and exhaled carbon monoxide concentration when the anonymity and confidentiality of respondents were guaranteed^36^. In addition, the majority of Malaysian youth were enrolled in government primary and secondary schools, which enables the findings of the study to be generalized to the majority of youth in Malaysia.

## CONCLUSIONS

The prevalence of dual/poly users was still low among Malaysian school-going adolescents. However, their characteristics were similar to those of mono users of tobacco products, and the majority used e-cigarettes before using other tobacco products. Therefore, proactive measures such as limiting access to e-cigarettes and identifying those non-tobacco users who potentially will use tobacco products should be implemented.

## Data Availability

The data supporting this research are available from the authors on reasonable request.
